# A Rare Presentation of Primary Frontotemporal Cerebral Lymphoma: A Case Report

**DOI:** 10.7759/cureus.61219

**Published:** 2024-05-28

**Authors:** Ahmed BenSghier, Meriem Bouabid, Soumiya Samba, Nourelhouda Mouhib, Soufiane Berhili, Mohamed Moukhlissi, Loubna Mezouar

**Affiliations:** 1 Department of Radiation Oncology, Centre Hospitalier Universitaire Mohammed VI, Oujda, MAR; 2 Department of Radiation Oncology, Faculty of Medicine and Pharmacy, Centre Hospitalier Universitaire Mohammed VI, Oujda, MAR; 3 Department of Radiation Oncology, Faculty of Medicine and Pharmacy, Mohammed First University, Oujda, MAR; 4 Department of Radiotherapy, Centre Hospitalier Universitaire Mohammed VI, Oujda, MAR

**Keywords:** case report, radiotherapy, chemotherapy, brain, lymphoma

## Abstract

Primary central nervous system (CNS) lymphoma is a rare and aggressive form of extranodal non-Hodgkin's lymphoma, limited to the brain, eyes, spinal cord, or leptomeninges without systemic involvement. This group of malignant tumors is characterized by a particular diagnostic, therapeutic, and evolutionary profile compared to other types of non-Hodgkin's lymphomas. We report a case of a young patient treated in our university hospital center for primary cerebral lymphoma who benefited from primary chemotherapy and then consolidation radiotherapy with good disease control and good tolerance.

## Introduction

Central nervous system (CNS) lymphomas present in two forms: primary CNS lymphomas and CNS lymphomas secondary to systemic involvement. Primary cerebral lymphoma is a rare variant of extranodal lymphomas, it accounts for almost 2% of all brain tumors and between 1% and 2% of all lymphomas. It is characterized by a rapid evolution and a less favorable prognosis than other lymphoma locations. High-dose chemotherapy supported by autologous stem cell transplantation remains the most used therapeutic strategy. Radiotherapy has its place in patients refractory to chemotherapy and sometimes in consolidation after primary chemotherapy [1]. We report a clinical case of primary cerebral lymphoma treated at the oncology center of OUJDA, Morocco.

## Case presentation

A 36-year-old patient, house painter, chronic smoker with a 20-pack-year history, occasional alcohol and cannabis user, had a sister who died of cancer, with no other notable medical history. He presented with headaches, not relieved by analgesic treatment lasting for six months, associated with episodes of morning vomiting, without neurological signs of deficit, and deterioration in general condition. The clinical examination found a conscious patient in good general condition with no detectable abnormality, particularly in the neurological, lymph node, and ophthalmological examination. The headaches appeared for the first time approximately six months before his admission to our hospital center associated with episodes of vomiting, treated by self-medication. The symptoms gradually worsened over over weeks, which led the patient to consult a family doctor who referred him to our hospital.

**Figure 1:**
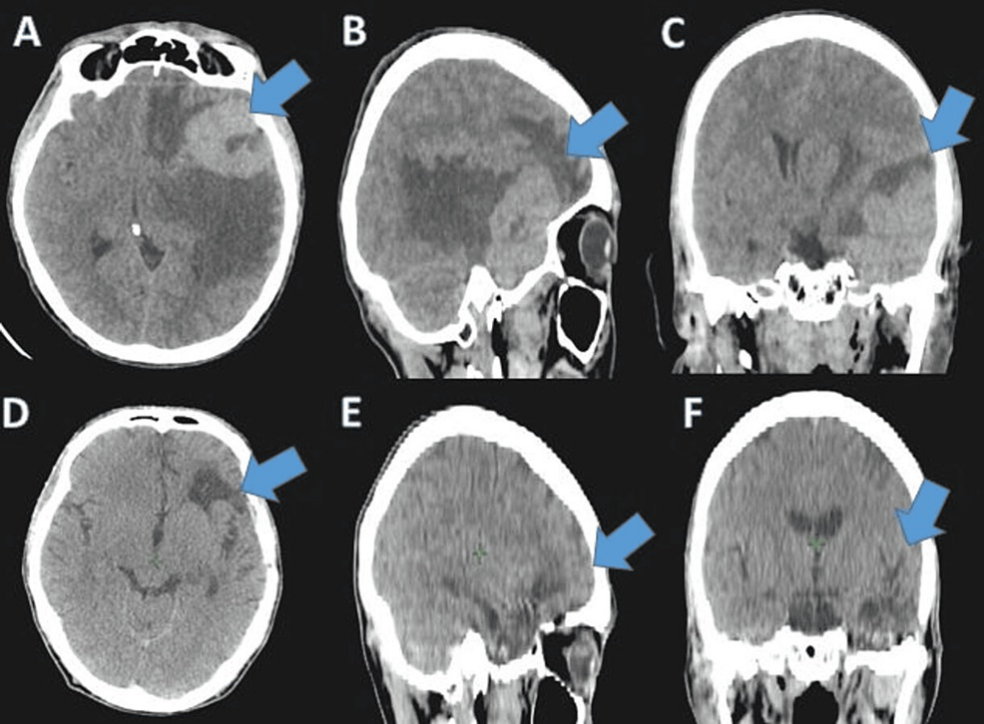
CT in (A, D) axial , (B, E) sagittal, and (C, F) coronal sections showing a left frontotemporal process (A-C) before and (D-F) after chemotherapy.

Brain computed tomography (CT) showed a spontaneously hyperdense left frontotemporal lesion with significant perilesional edema and mass effect on the lateral ventricle and midline structures (Figure 1).

**Figure 2:**
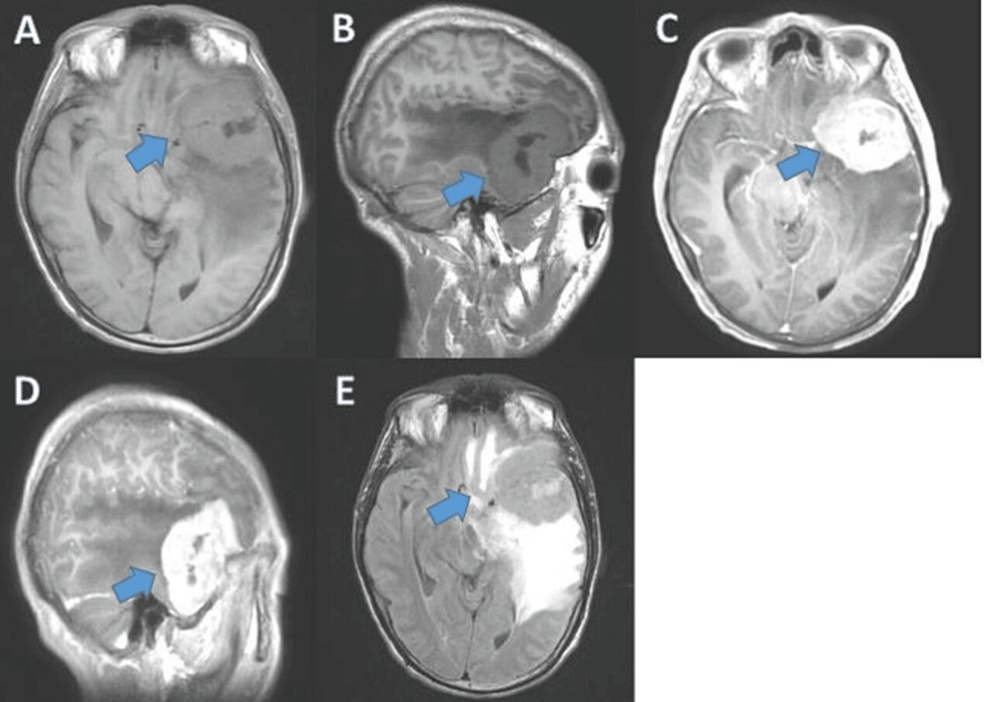
Brain MRI images: T1 sequence in axial section (A), sagittal section (B), injected axial section (C), and injected sagittal section (D); and T2 sequence in axial section (E), showing a left extra-axial frontotemporal process. MRI, magnetic resonance imaging

Brain magnetic resonance imaging (MRI) revealed the presence of a left extra-axial frontotemporal tumor process measuring 63 mm × 52 mm × 48 mm, with T1 hypo-signal, T2 hyper-signal, and diffusion, showing areas of marked hyper-signal on T2 and fluid-attenuated inversion recovery (FLAIR) sequences. The tumor was heterogeneously enhanced after the injection of contrast material and was surrounded by significant peri-lesional edema, suggestive of a glial tumor (Figure 2).

**Figure 3:**
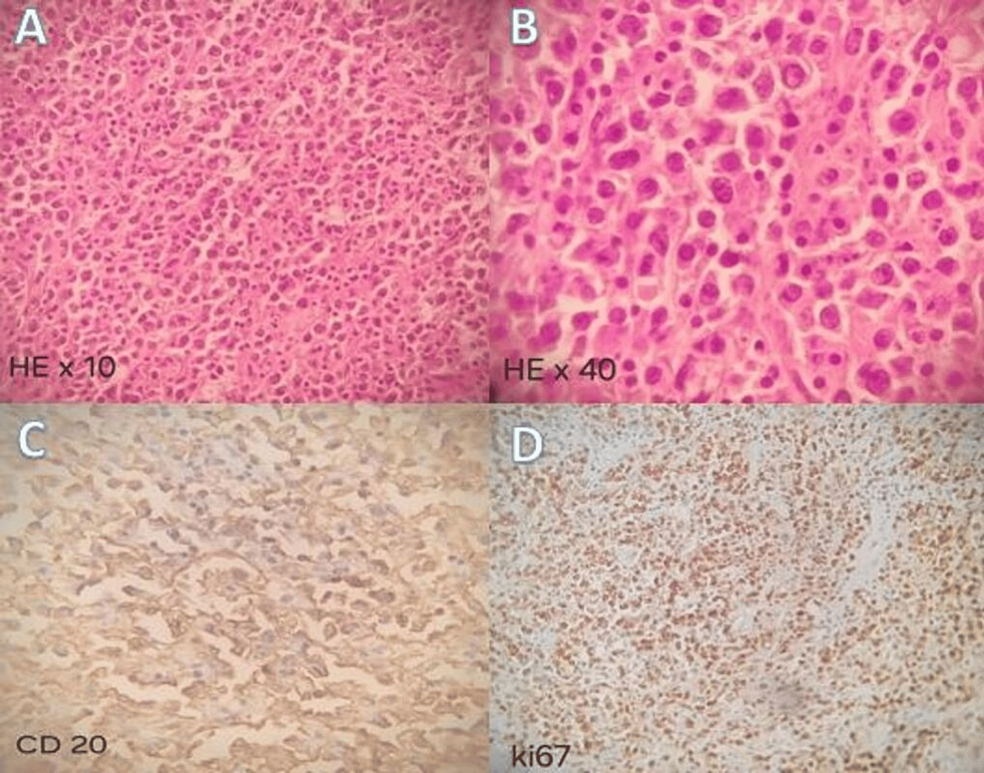
Histological and immunohistochemical appearance of the surgical biopsy. Hematoxylin and eosin staining at (A) x10 and (B) x40 magnification. Diffuse positivity of tumor cells to (C) anti-CD 20 and (D) anti-KI67 antibodies.

A surgical biopsy of the tumor was performed. Histological and immunohistochemical analysis indicated a non-germinal center type diffuse large cell non-Hodgkin B cell lymphoma, showing notable expression of the CD20 and MUM1 antigens by the tumor cells (Figure 3).

Cervico-thoraco-abdomino-pelvic CT and osteo-medullary biopsy did not show any suspicious lesion elsewhere, confirming the primary appearance of cerebral lymphoma.

The patient received four cycles of chemotherapy according to the MATRIX protocol without thiotepa, followed by consolidation radiotherapy to the entire brain at a dose of 36 Gy delivered in 18 fractions of 2 Gy each using three-dimensional conformal radiotherapy.

The patient tolerated the chemotherapy and radiotherapy treatment well. A good post-chemotherapy evolution was observed with a disappearance of the clinical symptoms and a complete regression of the tumor on the evaluation CT (Figure 1). After a three-month follow-up, the patient is alive and his disease is well controlled.

## Discussion

Primary cerebral lymphoma is a type of non-Hodgkin's lymphoma that affects the brain in isolation. While rare, its incidence is constantly increasing. It represents 1% to 2% of lymphomas and 2% of brain tumors, with a median age of discovery typically falling between 55 and 65 years [2]. The direct cause of this pathology remains unknown; however, it is established that its development can be favored by immune deficiency, regardless of its origin, such as HIV infection or others. Currently, the majority of cerebral lymphomas nonetheless occur in immunocompetent patients [2]. The clinical representation of primary cerebral lymphomas is nonspecific, with symptoms such as intracranial hypertension syndrome, focal neurological deficit, and cognitive disorders or, more rarely, epileptic seizures. Generally, the disorders appear in a rapidly progressive manner over a few days to a few weeks without notable general signs [3].

Brain MRI suggests the diagnosis of a cerebral lymphoma. The lesion appears as hypo- or iso-signal on T1 and hyper-signal on T2, often accompanied by varying degrees of peri-lesional edema. However, there are many atypical forms [4]. The decrease in N-acetyl-aspartate and the elevation in choline, along with the presence of an elevated lipid peak, are spectroscopic signs strongly suggestive of a lymphomatous brain lesion.

Despite advances in imaging, the diagnosis of cerebral lymphoma remains histological with immunohistochemical analysis of the collected material. In 80% to 90% of cases, it is a diffuse large B-cell lymphoma, high grade of malignancy. Once the diagnosis of a cerebral lymphoma is confirmed, it is necessary to carry out an extension assessment by a throraco-abdomino-pelvic scanner, an osteo-medullary biopsy, and possibly a positron emission tomography (PET) scan to eliminate other localizations of the lymphoma and confirm the primary nature of the disease. An HIV serology test must also be carried out with an ophthalmological examination since there is often a concomitant ophthalmological disease [5].

The treatment of primary cerebral lymphomas is primarily based on chemotherapy based on high doses of methotrexate, a molecule active on lymphoma cells with better cerebral bioavailability. Treatment is very restrictive because methotrexate is highly nephrotoxic, requiring intravenous hyperhydration and prolonged monitoring in a hospital setting.

Polychemotherapy with the addition of alkylating agents or cytarabine can significantly improve the initial response rate in young patients in good general health. Rituximab, a monoclonal antibody targeting the B-cell surface antigen CD20, resulted in significant improvement in response and clinical outcomes in cerebral diffuse large B-cell lymphoma [6]. Indeed, rituximab has been integrated into first-line therapeutic protocols for primary cerebral lymphomas.

Apart from the diagnostic interest, surgery had no place. Currently, total or subtotal surgical resection is taking up more and more space in the therapeutic arsenal of primary cerebral lymphoma; however, its benefit lacks sufficient scientific evidence [5].

Radiotherapy serves as a consolidation or salvage treatment in the event of relapse or partial tumor response, but it has side effects, especially cognitive disorders in people over 65 years of age. Some studies have shown that consolidation brain irradiation after primary chemotherapy improves progression-free survival without impact on overall survival, with a risk of neurotoxicity. However, the use of hyper-fractionated whole-brain radiotherapy could improve neurological tolerance [7]. Total doses of 30 to 36 Gy in classic fractionation are generally delivered. The irradiation volume includes the entire brain up to C1-C2, without forgetting the posterior part of the orbit and the eyeball [8]. When radiotherapy is used exclusively in fragile patients not eligible for high-dose methotrexate-based chemotherapy regimens, they receive doses of 40 Gy in 20 fractions over the entire brain, with additional irradiation of 20 Gy in 10 fractions on the tumor.

Cerebral lymphoma is a serious disease; however, it is typically highly responsive to treatment and potentially curable. Once complete remission has been achieved, monitoring is necessary to detect possible relapses. Regarding prognostic factors, advanced age, absence of surgical resection, and lack of radiotherapy or chemotherapy were all identified as poor prognostic factors [9].

## Conclusions

Primary CNS lymphoma is a rare entity, generally associated with a poor prognosis. Brain biopsy is necessary to confirm the diagnosis despite the development of radiological techniques. Currently, initial methotrexate-based chemotherapy is considered the standard of care for primary cerebral lymphomas. However, consolidation radiotherapy is not always recommended due to the risk of neurotoxicity.
